# Prevalence of Undiagnosed Depression and Its Sociodemographic and Clinical Correlates Among Adult Patients Attending a Medical Outpatient Department

**DOI:** 10.7759/cureus.93752

**Published:** 2025-10-03

**Authors:** Muhammad Javed, Muhammad A Rana, Mujtaba H Siddiqui, Abdalla Gamal Abdalla Afifi, Muhammad Mansoor Hafeez

**Affiliations:** 1 Internal Medicine, Services Institute of Medical Sciences, Lahore, PAK; 2 Critical Care Medicine, Bahria International Hospital, Lahore, PAK; 3 Internal Medicine, Akhtar Saeed Medical and Dental College, Lahore, PAK; 4 General Practice, Specialized Medical Center (SMC) Hospital, Riyadh, SAU; 5 Emergency, Bahria International Hospital, Lahore, PAK

**Keywords:** depression, hospitalizations, psychological, sociodemographic, undiagnosed

## Abstract

Background: Depression is a prevalent and debilitating mental health disorder that is often undiagnosed, particularly in medical outpatient departments (OPDs), where patients may present with somatic symptoms.

Objective: This study aims to assess the prevalence of undiagnosed depression and explore its sociodemographic and clinical correlates in adult patients attending an OPD.

Methods: This cross-sectional study was conducted at Bahria International Hospital, Lahore, Pakistan, from June 2024 to December 2024. A total of 350 adult patients were included in the study. Data were collected using a structured, pre-tested questionnaire that included both sociodemographic and clinical information. Sociodemographic data collected included age, gender, marital status, education level, employment status, and socioeconomic status.

Results: The study found that 125 (35.7%) participants had clinically significant depression, with 56 (44.8%) of these cases being undiagnosed. Sociodemographic factors such as lower socioeconomic status (p = 0.04) and marital status (divorced/widowed) (p = 0.02) were significantly associated with undiagnosed depression. Hypertension (p = 0.02) and diabetes (p = 0.01) were also significant predictors of undiagnosed depression. Multivariate logistic regression analysis revealed that female gender (OR = 1.45, p = 0.04), lower education levels (OR = 2.10, p = 0.02), and the presence of two or more chronic conditions (OR = 1.75, p = 0.02) were independently associated with undiagnosed depression.

Conclusion: The findings highlight the high prevalence of undiagnosed depression in medical OPD patients, particularly among those with chronic medical conditions. Routine screening for depression in OPDs, especially for patients with chronic diseases, is essential to ensure early detection and improve patient outcomes. Further research is needed to explore the long-term impact of undiagnosed depression and to develop targeted interventions for high-risk groups.

## Introduction

Depression is a very common mental illness that is debilitating and greatly affects the emotional, physical, and social condition of individuals. It causes a significant burden of disease on the world, as it is prevalent among the adult population across the world [1]. Nonetheless, depression remains common without being diagnosed, especially in hospital outpatient departments (OPDs), where patients usually report somatic symptoms that mask the psychological signs of their problems. The lack of proper diagnosis of the issue of depression in medical establishments is alarming because depression that is not treated can worsen the physical conditions, make it more difficult to treat them, and lead to a worse health outcome with that disorder [2].

Depression is a multifactorial condition, which is signified by consistent sadness, loss of interest in activities, tiredness, sleep disturbances, and eating disturbances, among others [3]. These symptoms are mostly psychological, but in most instances, they present themselves as physical complaints like constant pain, gastric problems, and headaches that are very common in the medical OPDs [4]. This overlap could make health care professionals concentrate on helping its victims over physical symptoms, leaving the existing depressive condition unidentified and unmanaged. Diagnosis of depression in these environments cannot be underrated, as undertreated depression poses a greater risk of emerging into chronic illnesses such as cardiovascular disease, diabetes, and hypertension, which are commonly treated in medical clinics [5]. 

Undiagnosed depression can worsen the prognosis of existing medical conditions. As an example, patients with diabetes or hypertension might have less adherence than others to their treatment regimens since they have depressive symptoms of fatigue, apathy, and lack of motivation. It, in its turn, may result in worse management of a disease and hospitalizations. Moreover, having depression is also known to cause the development of comorbid conditions, and those experiencing chronic conditions would be more likely to experience depression since they have to deal with their poor health (this takes a toll on their mind) [6].

The failure to diagnose depression can easily be judged by different sociodemographic and clinical factors. Research has indicated that gender, age, marital status, and socioeconomic status are some of the sociodemographic variables that can contribute to the identification and treatment of depression. An example of the first one is that women are more likely to be diagnosed with depression than men, which may be related to a higher help-seeking style and emotional symptoms. Conversely, men might be underreporting emotional issues, and as a result, depression will not be recognized among them [7]. Moreover, the lower socioeconomic groups tend to have limited access to mental healthcare due to the inability to access necessary resources, stigma about mental illness, and cultural norms that prevent the pursuit of professional psychological help in the case of psychological distress [8].

Another factor that seems to play a significant role is age, as depression in the case of elderly people may be neglected because of the numerous chronic diseases. The depression symptoms can be confused with the symptoms of normal aging, or medical professionals can believe that mood disorders are symptoms of physical illnesses that are more prevalent in elderly groups. By contrast, younger adults might be better able to report their symptoms based on mood, and, hence, depression might be more easily detected in their age category [9]. Clinical conditions and comorbidities can also increase the chances of depression being discovered. Chronic patients of a primary disease, whether it is heart disease, diabetes, or cancer, can suffer from the symptoms of depression, though they will be dwarfed by the physical symptoms of the primary disease [10].

Health care professionals might concentrate their attention on treating the medical condition and overlook the factor of depression as a reason leading to the overall deterioration of a patient. Polypharmacy in patients with multiple comorbidities can complicate the diagnosis of depression, as medications for physical illnesses may interact with antidepressants or exacerbate depressive symptoms [11]. Healthcare systems also exert a critical effect on the underdiagnosis of depression. Failure to provide healthcare professionals with comprehensive training in mental health, especially in the primary care and medical OPD, may expose one to the risk of missing the opportunity to identify and treat depression early [12].

This study aims to assess the prevalence of undiagnosed depression and explore its sociodemographic and clinical correlates among adult patients attending a medical outpatient department.

## Materials and methods

This cross-sectional study was conducted at Bahria International Hospital, Lahore, Pakistan, from June 2024 to December 2024. A total of 350 adult patients were included in the study. The sample size of 350 was calculated using a single-proportion formula, assuming an expected prevalence of depression of 30% from prior studies, with a 95% confidence level and a 5% margin of error. The sample size was determined based on a power calculation to ensure sufficient statistical power for detecting significant associations between depression and sociodemographic and clinical factors. The inclusion of 350 patients ensures the results are representative of the outpatient population, increasing the generalizability of the findings. The study included adults aged 18 years and older who were attending the medical outpatient department during the study period and were willing to provide informed consent for participation. Patients were excluded if they had a known history of psychiatric disorders such as major depression, bipolar disorder, or schizophrenia before the study. Individuals who were unable or unwilling to participate due to language barriers or cognitive impairments were also excluded. Patients who were already undergoing psychiatric treatment at the time of the study were not considered eligible.

Data collection

Data was collected using a structured, pre-tested questionnaire that included both sociodemographic and clinical information. Sociodemographic data collected included age, gender, marital status, education level, employment status, and socioeconomic status. Clinical data included details about the patient's medical history, comorbidities, medication use, and the severity of any chronic diseases they were managing. Depression was assessed using the Patient Health Questionnaire-9 (PHQ-9) [13], a widely used screening tool for diagnosing depression in primary care settings. The PHQ-9 consists of nine questions regarding the presence and severity of depressive symptoms experienced in the past two weeks, and it has been validated in various clinical settings, including OPDs. A score of 10 or higher on the PHQ-9 indicates the presence of clinically significant depression. After obtaining informed consent, participants were asked to complete the questionnaire, including the PHQ-9 screening tool. A trained research assistant administered the questionnaire to ensure that participants understood the questions. All patient information was kept confidential, and no personal identifiers were recorded in the study database. Once the PHQ-9 results were collected, patients identified with clinically significant depression (a score of 10 or higher) were further evaluated by the research team to determine whether they had previously been diagnosed with depression by a healthcare professional. Those who reported no prior diagnosis but screened positive were classified as “previously unrecognized cases of depression.

Data was analyzed using SPSS version 26.0 (IBM Corp., Armonk, NY). Descriptive statistics (mean, standard deviation, frequencies, and percentages) were used to summarize sociodemographic and clinical characteristics of the study participants. The prevalence of undiagnosed depression was calculated by dividing the number of patients with undiagnosed depression by the total number of participants. Multivariate logistic regression analysis was conducted to assess the independent predictors of undiagnosed depression, adjusting for potential confounders such as age, gender, and medical comorbidities. The model was adjusted for age, gender, marital status, education level, socioeconomic status, and presence of chronic conditions (hypertension, diabetes, cardiovascular disease, chronic respiratory disease, and osteoarthritis). A p-value <0.05 was considered statistically significant.

## Results

Data were collected from 350 patients; 56 (16%) screened positive, while 294 (84%) fell into the previously diagnosed/screened negative category. Males represented 44.3% of the total sample, with a notably higher share of positivity (29.7%) compared to females (17.9%), while the majority of those in the previously diagnosed/screened negative group were female (62.9%). Most participants were married (68%), and within this group, 17.6% screened positive, whereas singles, divorced, and widowed individuals showed lower proportions of positivity. Education level revealed a relatively even distribution of screening positivity across primary (32.1%), secondary (41.1%), and university (26.8%) categories. Socioeconomic status indicated that the lower-middle group (60%) not only comprised the largest portion of the sample but also contributed most to the positives (57.1%). Regarding chronic disease duration, positivity was similarly distributed across 0-5 years (35.7%), 6-10 years (39.3%), and more than 10 years (25%) (Table 1).

**Table 1:** Demographic and Sociodemographic Characteristics of Participants Demographic and Sociodemographic Characteristics of Participants. Data are presented as frequency (n) and percentage (%).

Characteristic	Total (N=350)	Screened Positive (n=56)	Previously Diagnosed/Screened Negative (n=294)
Gender			
Male	155 (44.3%)	46 (29.7%)	109 (37.1%)
Female	195 (55.7%)	10 (17.9%)	185 (62.9%)
Marital Status			
Married	238 (68.0%)	42 (17.6%)	196 (66.7%)
Single (never married)	56 (16.0%)	8 (14.3%)	48 (16.3%)
Divorced	28 (8.0%)	4 (7.1%)	24 (8.2%)
Widowed	28 (8.0%)	2 (3.6%)	26 (8.8%)
Education			
Primary	105 (30.0%)	18 (32.1%)	87 (29.6%)
Secondary	140 (40.0%)	23 (41.1%)	117 (39.8%)
University	105 (30.0%)	15 (26.8%)	90 (30.6%)
Socioeconomic Status			
Lower-middle	210 (60.0%)	32 (57.1%)	178 (60.5%)
Middle	105 (30.0%)	18 (32.1%)	87 (29.6%)
Upper-middle	35 (10.0%)	6 (10.7%)	29 (9.9%)
Duration of Chronic Disease			
0–5 years	123 (35.1%)	20 (35.7%)	103 (35.0%)
6–10 years	140 (40.0%)	22 (39.3%)	118 (40.1%)
>10 years	87 (24.9%)	14 (25.0%)	73 (24.8%)

Chronic medical conditions were prevalent among the study participants, with 123 (35.1%) of the total population having hypertension, 98 (28%) diabetes mellitus, and 63 (18%) cardiovascular disease. Diabetes mellitus was reported in 98 patients (28.0%), with a notably higher prevalence among those screening positive (38.4%) versus negative (22.2%). Cardiovascular disease affected 63 participants (18.0%), again showing higher rates in the screened positive group (22.4%) compared to the screened negative group (15.6%). Chronic respiratory disease was identified in 28 cases (8.0%) and osteoarthritis in 18 cases (5.1%), with both conditions occurring slightly more often among individuals screening positive (9.6% and 5.6%, respectively) than those screening negative (7.1% and 4.9%, respectively) (Table 2).

**Table 2:** Prevalence of Chronic Medical Conditions Data are presented as frequency (n) and percentage (%).

Condition	Total (N=350)	Screened Positive (n=125)	Screened Negative (n=225)
Hypertension	123 (35.1%)	56 (44.8%)	67 (29.8%)
Diabetes mellitus	98 (28.0%)	48 (38.4%)	50 (22.2%)
Cardiovascular disease	63 (18.0%)	28 (22.4%)	35 (15.6%)
Chronic respiratory disease	28 (8.0%)	12 (9.6%)	16 (7.1%)
Osteoarthritis	18 (5.1%)	7 (5.6%)	11 (4.9%)

The logistic regression analysis showed that several factors were independently associated with screening positive for depression. It was significantly higher in females (OR = 1.45, p = 0.04), indicating a gender disparity. Education level was another significant factor, with those with primary education showing higher odds of undiagnosed depression compared to university-educated individuals (OR = 2.10, p = 0.02). Socioeconomic status was also a predictor, with lower-middle-class individuals being more likely to have undiagnosed depression compared to those from upper-middle-class backgrounds (OR = 1.85, p = 0.04).

**Table 3:** Logistic Regression Predictors of Screening Positive Data are presented as odds ratio (OR) with 95% confidence interval (CI).

Predictor	Odds Ratio (OR)	95% Confidence Interval (CI)	Wald χ²	p-value
Gender (female vs male)	1.45	1.02 – 2.06	4.18	0.04
Education (primary vs university)	2.10	1.32 – 3.32	5.46	0.02
Socioeconomic status (lower-middle vs upper-middle)	1.85	1.06 – 3.23	4.12	0.04
Chronic conditions (two or more vs one)	1.75	1.12 – 2.62	5.34	0.02
Marital status (divorced/widowed vs married)	2.10	1.11 – 3.99	4.22	0.02

The severity of depression was significantly associated with chronic conditions such as hypertension and diabetes. Among those with mild depression, 9 (20%) had hypertension, while this figure increased to 14 (45.2%) in those with severe depression (p = 0.04). Similarly, 10 (22.2%) of patients with mild depression had diabetes, compared to 14 (45.2%) of those with severe depression (p = 0.02). Although cardiovascular disease and chronic respiratory disease showed a trend toward higher depression severity, these associations were not statistically significant (p > 0.05).

**Table 4:** Severity of Depression and Its Association With Chronic Conditions Data are presented as frequency (n) and percentage (%). p-values were calculated using the chi-square (χ²) test.

Chronic Condition	Mild Depression (n=45)	Moderate Depression (n=34)	Severe Depression (n=31)	χ² (df)	p-value
Hypertension	9 (20.0%)	12 (35.3%)	14 (45.2%)	χ²=6.37 (2)	0.04
Diabetes mellitus	10 (22.2%)	11 (32.4%)	14 (45.2%)	χ²=7.95 (2)	0.02
Cardiovascular disease	5 (11.1%)	7 (20.6%)	9 (29.0%)	χ²=4.81 (2)	0.09
Chronic respiratory disease	3 (6.7%)	3 (8.8%)	5 (16.1%)	χ²=2.10 (2)	0.35
Osteoarthritis	2 (4.4%)	2 (5.9%)	3 (9.7%)	χ²=1.04 (2)	0.59

## Discussion

The current study evaluated the rate of undiagnosed depression of adult patients visiting a medical OPD and identified factors related to sociodemographic and clinical characteristics associated with undiagnosed depression. The results of this research make several meaningful suggestions regarding the underrecognition of depression in a medical context, the influence of chronic medical diseases, sociodemographic factors, and the usage of medication on the possibility of undiagnosed depression. It was shown that 35.7% participants were found to have clinically significant depressive symptoms, with a PHQ-9 score of 10 or greater. Out of these numbers, 44.8% had not received a depression diagnosis before by a healthcare professional. This shows that there was an aspect of undiagnosed depression in almost 16% of the total study population. These findings agree with Seifu et al.'s findings that have found a high percentage of undiagnosed depression in the medical institutions [14]. 

In the analysis, a number of sociodemographic factors were found to have significant relationships with depression that remained undiagnosed. The prevalence of undiagnosed depression was higher among female patients than male patients, but that was not statistically significant (p = 0.11) [15]. It can be assumed that the elevated rates of undiagnosed depression in females can be attributed to gender differences in presenting the symptoms. Women tend to report having more emotional distress and seek help with their psychological manifestations, but men may underreport depression or fail to request mental health issues because of social or cultural expectations [16]. The results are in line with the research of Kuehner (2017), according to which depression is more common among women but could be unrecognized in men because they could experience more somatic symptoms [17]. Marital status also contributed to the likelihood of screening positive for depression, with the highest prevalence observed among divorced and widowed participants in this study. This finding is consistent with prior research showing that individuals who are divorced or widowed are more vulnerable to depression due to factors such as emotional distress, social isolation, and financial strain. This observation concurs with prior studies that they are more susceptible to depression by the divorced or widowed individuals as these situations usually entail emotional, social, and financial stress. On the same line of reasoning, low education levels and low socioeconomic status were linked with increased levels of undiagnosed depression. These findings concur with the literature in terms of the possibility that lower socio-economic members of society might experience emotional care service barriers such as stigmatization, low awareness, and access to medical services related to depression, which makes them unable to access or approach proper care of depression [17-19].

Chronic medical conditions, particularly hypertension and diabetes, also emerged as important factors in this study, with a higher proportion of patients screening positive for depression reporting these conditions compared to those screening negative. These findings are consistent with previous studies that have documented a strong comorbidity between chronic diseases and depressive symptoms, suggesting a bidirectional relationship in which each condition can worsen the course of the other [19-21]. As an illustration, feelings of disinterest, fatigue, and difficulty concentrating are common depressive symptoms that may reduce treatment adherence and thereby worsen chronic illnesses [22]. In the present study, the empirical analysis further showed that patients with severe depressive symptoms were more likely to have chronic medical conditions, particularly hypertension and diabetes. This pattern is consistent with previous reports highlighting the strong association between depression severity and comorbid chronic diseases [23-24].

This study has several limitations that should be acknowledged. First, as a single-center cross-sectional study, the findings may not be fully generalizable to other healthcare settings or populations across Pakistan. Second, the reliance on the PHQ-9 screening tool, although validated, may introduce reporting bias since depressive symptoms were self-reported and could be under- or overestimated by participants. Third, the exclusion of patients with pre-diagnosed psychiatric conditions or those already receiving treatment may have led to an underrepresentation of the true burden of depression in the outpatient setting.

## Conclusions

It is concluded that undiagnosed depression is highly prevalent among adult patients attending medical outpatient departments, particularly in those with chronic medical conditions such as hypertension and diabetes. The study reveals that significant sociodemographic factors, including gender, marital status, education level, and socioeconomic status, play a crucial role in the likelihood of depression remaining undiagnosed. These findings highlight the urgent need for routine depression screening in medical settings, especially for patients with chronic diseases.
